# African schistosomiasis in mainland China: risk of transmission and countermeasures to tackle the risk

**DOI:** 10.1186/1756-3305-6-249

**Published:** 2013-08-28

**Authors:** Wei Wang, You-Sheng Liang, Qing-Biao Hong, Jian-Rong Dai

**Affiliations:** 1 Jiangsu Institute of Parasitic Diseases, 117 Yangxiang, Meiyuan, Wuxi, Jiangsu Province 214064, People’s Republic of China; 2 Key Laboratory on Technology for Parasitic Disease Prevention and Control, Ministry of Health, 117 Yangxiang, Meiyuan, Wuxi, Jiangsu Province 214064, People’s Republic of China; 3 Jiangsu Provincial Key Laboratory of Molecular Biology of Parasites, 117 Yangxiang, Meiyuan, Wuxi, Jiangsu Province 214064, People’s Republic of China

**Keywords:** African schistosomiasis, *Biomphalaria straminea*, Imported case, Transmission risk, Research priority, China

## Abstract

Schistosomiasis is a major disease of public health importance in humans occurring in 76 countries of the tropics and sub-tropics. In China, schistosomiasis japonica is one of the highest priorities in communicable disease control defined by the central government. Since 1970s, the habitats of *Biomphalaria straminea*, an intermediate host of *Schistosoma mansoni* in South America, have been identified in Hong Kong Special Administrative Region and Shenzhen city, Guangdong province of China. With the sharp growth in the China-aided projects in Africa and labor services export to Africa, a gradual rise in the cases infected with *S. haematobium* or *S. mansoni* is reported in those returning from Africa to China. The existence of intermediate snail hosts and import of infectious source of schistosomiasis results in concern about the transmission of African schistosomiasis in mainland China in the context of global climate change. This paper evaluates the risk of transmission of African schistosomiasis in China, and proposes countermeasures and research priorities to tackle the risk.

## Review

Schistosomiasis is a snail-borne parasitic disease caused by trematodes of the genus *Schistosoma*, which affects more than 207 million people in 76 countries of the tropical and subtropical regions [1]. Six species of the blood fluke are reported to infect humans causing schistosomiasis, including *S. haematobium*, *S. japonicum*, *S. mansoni*, *S. intercalatum*, *S. mekongi* and *S. malayensis*; *S. mansoni*, *S. japonicum* and *S. haematobium* are the most significant species for human disease but vary in geographical distribution. The transmission of this neglected tropical disease is determined by the existence and geographic distribution of the intermediate host snails (Table 1). It has been proved that *Schistosoma* is endemic in regions where intermediate host snails are identified, while the transmission does not occur in areas in absence of host snails, although imported schistosomiasis cases are detected [2].

**Table 1:** Parasite species, intermediate hosts and geographic distribution of the disease

**Parasite species**	**Intermediate hosts**	**Geographic distribution**
*S. mansoni*	Eighteen species of *Biomphalaria*, including *B. glabrata*, *B. alexandrina*, *B. pfeifferi*, *B. straminea*, *etc*.	Africa, the Middle East, the Caribbean, Brazil, Venezuela, Suriname
*S. japonicum*	*Oncomelania hupensis*	China, Indonesia, the Philippines
*S. haematobium*	*Bulinus* spp., including *B. truncatus*, *B. africanus*, *B. globosus*, *etc*.	Africa, the Middle East

In China, only *S. japonicum* is endemic. Since the 1970s, the snail intermediate hosts of *S. mansoni* have been found in the natural environments of Hong Kong Special Administrative Region (SAR) and Shenzhen city, Guangdong province in China [3,4], and high-density *Biomphalaria straminea* habitats have been identified in many rivers of Shenzhen city recently [5]. With a quickening pace of integration of the global economy, the deepening collaboration between China and African countries and Chinese rapid economic development, there has been a sharp growth in China-aided projects in Africa and labor services export to Africa, and a gradual increase in the cases infected with *S. haematobium* or *S. mansoni* is reported in those returning to China [6-8]. Once these infected cases, as sources of infection of schistosomiasis, are imported to regions where the snail intermediate hosts of African schistosomes are present, there is a high possibility of transmission of African schistosomiasis in China. This has received much attention. Hereby, we evaluated the risk of transmission of African schistosomiasis in China and proposed some countermeasures and research priorities to tackle the risk.

### Risk of transmission of African schistosomiasis in mainland China

#### Existence of snail intermediate hosts of African schistosomes in mainland China

The emergence and transmission of a snail-transmitted parasitic disease is governed by the geographic distribution of the snail hosts [2]. The existence of *Biomphalaria* spp. and *Bulinus* spp., the intermediate hosts of *S. mansoni* and *S. haematobium*, is a prerequisite for the transmission of schistosomiasis mansoni and haematobia. Eighteen species of *Biomphalaria* serve as intermediate hosts of *S. mansoni*, including *B. glabrata*, *B. alexandrina*, *B. pfeifferi*, *B. straminea*, *etc*. [9]. To compare the development of *S. mansoni* in *B. tenagophila*, *B. straminea* and *B.glabrata*, 200 snails of each species were individually exposed to 50 miracidia of the *S. mansoni* AL line, and it was found that the infection rates of the snails and the average numbers of cercariae shed per day were 32.6% and 79 ± 90 for *B. tenagophila*, 11.3% and 112 ± 100 for *B. straminea*, and 75.3% and 432 ± 436 for *B. glabrata*, respectively. The lower levels of infection and average numbers of cercariae shed by *B. tenagophila* and *B. straminea* are considered to be related to their more potent internal defense systems [10]. It was found that *B. tenagophila* was poorly compatible with the LE strain of *S. mansoni* (Frandsen’s total cercariae production index class II) and compatible with the SJ and AL strains (class III), and *B. straminea* was not very compatible with the SJ strain (class I) and poorly compatible with the LE and AL strains (class II), while *B. glabrata* was extremely compatible (class VI) with all the three lines of *S. mansoni*[11]. In addition, *B. straminea* and *B. tenagophila* from different Argentine localities displayed different susceptibility and compatibility to *S. mansoni* EC strain (class 0-II), whereas *B. orbigny* and *B. oligoza* were incompatible [12]. These studies indicate that different species of *Biomphalaria* vary in the susceptibility to various strains of *S. mansoni*.

In 1974, a snail intermediate host *B. straminea* of *S. mansoni* in South America, as an invasive snail species, was first discovered in a stream in Hong Kong [3]. This snail species was first found in some ponds, ditches and rivers of Shenzhen city, mainland China in 1981 [4], and a further survey in 1983 showed the wide distribution of *B. straminea* in Shenzhen river systems and demonstrated that the snails were spread into Shenzhen from Hong Kong via water [13], which proves that *B. straminea* is able to survive, reproduce, and form new populations naturally in southern China such as Hong Kong and Shenzhen, and it can spread along the river systems. A recent epidemiology survey revealed that *B. straminea* as a predominant snail population has widely spread in Shenzhen city, and many snail habitats had been observed [5]. The introduction of the intermediate host snails and their survival, reproduction, spread and formation of new habitats in natural environments of southern China constitutes the prerequisite for the transmission of schistosomiasis mansoni in China.

#### Continuous import of source of infection of schistosomiasis into mainland China

It is estimated that 85% of the world’s cases of schistosomiasis are in Africa, and at least 90% of those requiring treatment for schistosomiasis live in Africa [14]. Since 1970s when China started the program to aid African infrastructure construction and sent engineering technicians and workers to African countries, imported cases with *S. mansoni* or *S. haematobium* infections have been continuously detected in returners from Africa [15-28]. Table 2 demonstrates the imported cases with African schistosomiasis detected among returners from Africa in China. Although there is currently lack of knowledge on comprehensive epidemiological surveys of schistosome infections among laborers working in African countries, the available case reports prove the real existence of imported African schistosomiasis cases returning from Africa in China, which constitutes the necessary condition for the transmission of African schistosomiasis in mainland China. We summarized the characteristics of imported cases with African schistosomiasis according to the available data (see the following List of Saints). The uncertainty, mobility, and likelihood of development of praziquantel resistance in the imported cases with African schistosomiasis increase the complexity and difficulty of control of the imported infectious sources. It is therefore considered that there is a gradually increasing risk of transmission of African schistosomiasis in mainland China.

**Table 2:** Reported imported cases of African schistosomiasis in China

**Year**	**Location (province)**	**No. infections**	**Source of infection**	**Country where infections occur**	**Reference**
1979	Beijing	67	*S. mansoni*	Unreported	[15]
1980	Beijing	15	*S. haematobium*	Zanzibar, Tanzania, and Zambia	[16]
1984	Shaanxi	2	*S. haematobium*	Yemen	[17]
1988	Beijing	22	*S. haematobium*	Egypt and Mali	[18]
1991	Hubei	1	*S. haematobium*	Egypt	[19]
1992	Jilin	1	*S. haematobium*	Nepal	[20]
1992	Beijing	2	*S. haematobium*	South Africa and Zimbabwe	[21]
1992	Hubei	1	*S. haematobium*	Egypt	[22]
1992	Fujian	21	*S. haematobium*	Yemen	[23]
2001	Beijing	75	*S. mansoni*	Unreported	[24]
2005	Jiangsu	1	*S. haematobium*	Mozambique	[25]
2007	Shaanxi	1	*S. haematobium*	Angola	[26]
2008	Beijing	1	*S. mansoni*	Unreported	[27]
2009	Beijing	2	*S. mansoni*	Unreported	[28]
2010	Hunan	28	*S. haematobium*	Mozambique	[7]
2011	Hunan	184	*S. haematobium*	Angola, Mozambique, Zambia, Congo, Liberia, South Africa	[8]
2011	Beijing	2	*S. mansoni*	Ethiopia	[6]

**Figure 1:**
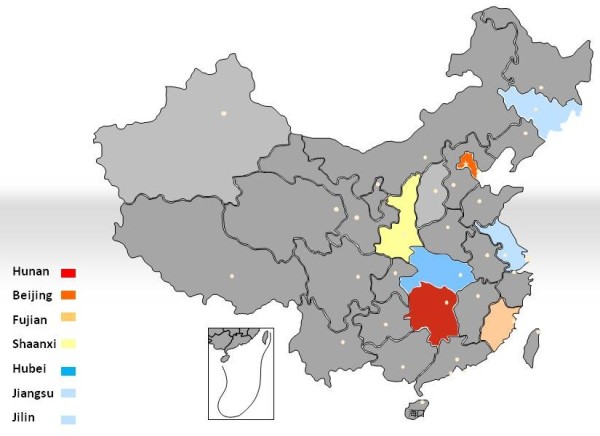
Distribution of imported cases with African schistosomiasis in China.

1. High schistosome infection rate in field workers and underestimation of actual cases. It has been shown that most of the subjects infected with African schistosomes are identified in field workers during physical examinations, due to high frequency of contact with the infested water. Since the cases infected with *S. mansoni* usually have mild or even no obvious symptoms, few seek medical services. It is therefore estimated that the actual infections are underestimated.

2. High proportion of missed diagnosis and misdiagnosis. Unlike *S. japonicum* infections, the clinical manifestations of schistosomiasis mansoni are comparatively milder, which are characterized by diarrhea, weakness and systemic ache. These non-specific symptoms are easily neglected, resulting in missed diagnosis. The major clinical manifestations of schistosomiasis haematobia involve hematuresis, bladder irritation, and urinary tract obstruction, which are often misdiagnosed as sexually transmitted diseases, cystitis, tuberculosis and tumors due to the lack of knowledge on diagnosis of the disease in Chinese clinicians.

3. Wide distribution and high mobility. We summarized the imported cases with African schistosomiasis reported previously in mainland China, and found that the patients were widely distributed in the country (Figure 1). A survey of 263 returners infected with *S. haematobium* in Africa revealed that the residency of these cases is distributed in 17 provinces of China (unpublished data).

4. Potential likelihood of development of praziquantel resistance. Since there are cases infected with *S. mansoni* and *S. haematobium* in whom standard treatment therapy fails to clear the infections reported, the emergence of praziquantel resistance in these imported cases should be concerned.

### Global climate change

The lifecycle of schistosomes includes two hosts: a definitive host where the parasite undergoes sexual reproduction, and a single intermediate snail host where there are a number of asexual reproductive stages [2]. The geographic distribution of intermediate host snails and the development of schistosome larvae within snails are closely associated with environmental temperature. The snail species has been shown to exhibit a high adaptability to humidity and temperature, however, various species of snails and the schistosomes parasitizing snail hosts have their respective optimum temperatures for survival and reproduction. *B. straminea* lives naturally in freshwater at tropical regions, with the optimum water temperature of 20–30°C for growth [29]. It is found that juvenile *B. straminea* snails grows quickly at 24°C, while a large number of snails start to die at 16–17°C during the daytime and at 7–8°C during the night [30], indicating that *B. straminea* survives in a temperature-dependent manner. Habitats are found to form 30 years after the first discovery of *B. straminea* snails in Shenzhen, southern China, demonstrating that the natural environment in Shenzhen is suitable for the survival and reproduction of this snail species. In addition, the environmental temperature is reported to directly affect snail egg hatching, juvenile snail growth, adult snail survival and matching, invasion of miracidia into snails, development of schistosome larvae within snails, and the release of cercariae from snails, which plays a crucial role in the transmission of schistosomiasis [31].

There is burgeoning consensus that global warming is real. According to the report of the Intergovernmental Panel on Climate Change, the Earth’s surface temperature is likely to increase, on average, by 1.4°C to 5.8°C over the period 1990 to 2100. This increase is about two to tenfold higher than the average temperature increase already observed during the 20th century [32]. It has been predicted, based on recent meteorological models using the mean annual temperature for the whole of China, that the mean temperature will continue to rise, indeed at an accelerated pace with predicted increases by 1.7°C in 2030 and by 2.2°C in 2050, respectively [33]. The continuous rise in the Earth’s surface temperature would certainly create an appropriate condition for the survival and reproduction of the intermediate host snails, as well as the development, parasitizing and transmission of schistosomes, and affect the original landscape of schistosomiasis, thereby increasing the risk of transmission of schistosomiasis.

### Countermeasures and research priorities to tackle the risk

**Figure 2:**
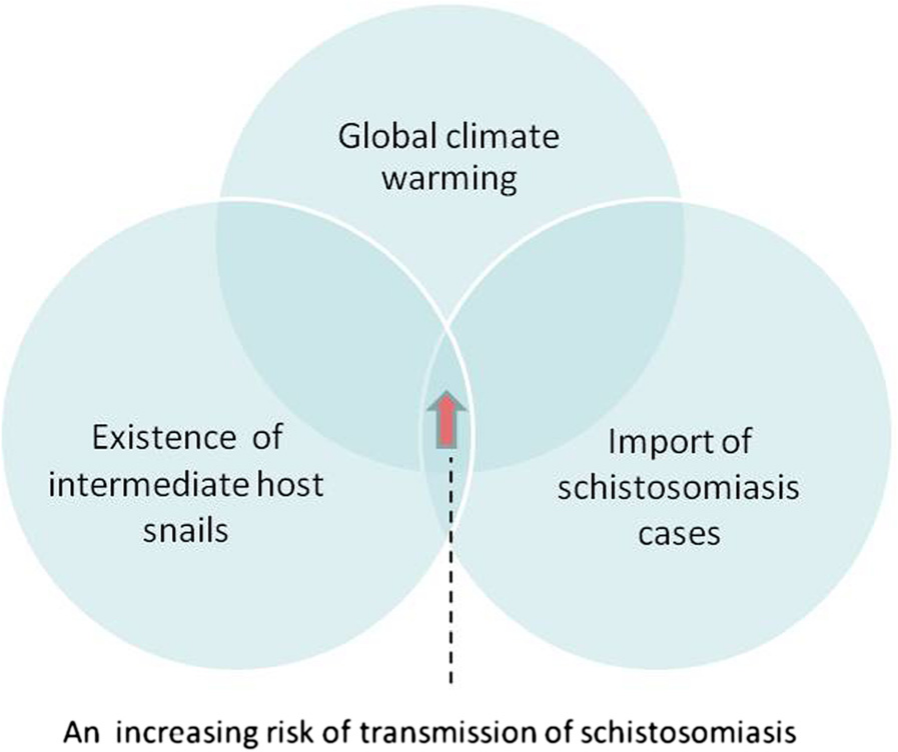
The continuous increase in the imported cases plus the existence of intermediate host snails would result in an increasing risk of transmission of African schistosomiasis in China in the context of global warming.

Considering that there are habitats of snail intermediate hosts of *S. mansoni* currently in China, the risk of transmission of African schistosomiasis in China continuously increases in the context of import of schistosomiasis cases as a source of infection into the country and global climate warming (Figure 2). The following interventions and research priorities are therefore proposed to reduce or eliminate the risk of transmission of imported African schistosomiasis in mainland China (see the List of Saints).

1. A systematic survey of schistosome infections in people returning from African countries and a comprehensive evaluation of the prevalence, transmission route and pattern of infection of schistosomiasis in those working in Africa currently are required, so as to develop an effective strategy to avoid the emergence of public-health concern in China.

2. A systematic investigation of freshwater mollusks with special consideration of snail intermediate hosts including *Biomphalaria* spp. and *Bulinus* spp. should be performed in Shenzhen, Hong Kong and the neighboring regions, to understand the species, geographic distribution and density of the mollusks and their correlations with the surrounding environments. Determination of the infectivity of the water body is also needed. Snail control interventions should be implemented in snail habitats to eliminate the reproduction and spread of the snail intermediate hosts.

3. Health education pertaining to schistosomiasis prevention and control, international travel healthcare and global status of schistosomiasis should be strengthened in those moving to Africa due to work, business and travel, and the booklets covering knowledge on distribution in Africa, harm, pattern of infection and preventive interventions of schistosomiasis are required to be compiled under the organization of health sections in collaboration with commercial and diplomatic sections, and are allocated before they go to Africa, so as to enhance their self-protection awareness and prevent the occurrence of infections. In addition, the detection and monitoring of schistosomiasis should be strengthened in populations returning from schistosome-endemic nations to China, and the entry-exit inspection and quarantine sections should put their emphases on consultation on prevention and control of schistosome infections and introduction of global status of schistosomiasis in addition to monitoring of infectious diseases.

4. Basic and operational studies such as determination of the susceptibility of *B. straminea* found in Shenzhen and Hong Kong to *S. mansoni*, observation on the growth and development of *S. mansoni* in *B. straminea*, and assessment of the susceptibility of the mature cercaria released from *B. straminea* to definitive hosts, should be conducted, and further studies to search for immunodiagnostic techniques for screening of *S. mansoni* and *S. haematobium* infections, and investigate the ecology and control of the snail intermediate hosts seem justified.

5. Since there are cases with schistosomiasis mansoni or haematobia returning from Africa in whom standard praziquantel treatment fails to clear the infections [34-38], the detection and monitoring of praziquantel resistance has to be enhanced in imported cases of African schistosomiasis to timely identify those infected with nonsusceptible or resistant schistosome isolates. Once reduced sensitivity to praziquantel or resistance is detected, other antischistosomal drugs as alternatives of praziquantel, are employed for treatment of human infections, which can effectively cure cases timely. On other hand, such a replacement could rapidly remove the resistant strains from the schistosome populations in a certain area, which would effectively control the spread of drug resistance-associated genes in the endemic foci [39].

## Conclusions

With a quickening pace of integration of global economy and Chinese rapid development of international trade, more and more China-aided projects in Africa and the continuous growth in labor service export would necessarily increase the probability of import of subjects infected with African schistosomes, as sources of infection, into China. In the context of global climate warming, the likelihood of introduction of the snail intermediate hosts into China and the subsequent spreading and expansion increases continuously, thereby breaking through the limitation of the original geographic distribution of the snail hosts. It is considered that the continuous growth in imported schistosomiasis cases will certainly increase the risk of transmission of African schistosomiasis in China in the presence of snail intermediate hosts. Based on epidemiological survey and basic and operational studies, assessment of the risk of transmission of African schistosomiasis and establishment of a surveillance-response system is critical to prevent the transmission.

## Competing interests

The authors declare that they have no conflicts of interest.

## Authors’ contributions

WW and YSL conceived and designed the review; WW, QBH and JRD conducted the review of the literature, extracted the pertinent data, and performed analysis of data. WW prepared the first draft of the manuscript; YSL provided strategic advice and assisted with editing of the manuscript. All authors read and approved the final version of the manuscript.

## References

[B1] Steinmann P Keiser J Bos R Tanner M Utzinger J Schistosomiasis and water resources development: systematic review, meta-analysis, and estimates of people at risk Lancet Infect Dis 2006 6 411 425 10.1016/S1473-3099(06)70521-7 16790382

[B2] Ross AG Bartley PB Sleigh AC Olds GR Li Y Williams GM McManus DP Schistosomiasis N Engl J Med 2002 346 1212 1220 10.1056/NEJMra012396 11961151

[B3] Meier-Brook C A snail intermediate host of *Schistosoma mansoni* introduced into Hong Kong Bull World Health Organ 1974 51 661 4549615 PMC2366262

[B4] Liu YY Wang YX Zhang WZ The discovery of *Biomphalaria straminea* (Dunker), an intermediate host of *Schistosoma mansoni*, from China Acta Zootaxonomica Sin 1982 7 256 (in Chinese)

[B5] Gao ST Li XH Huang SY Xie X Mei SJ Ruan CW Huang DN Primary investigation of distribution and ecological environment of *Biomphalaria straminea* in Dasha and Guanlan Rivers in Shenzhen areas Chin Trop Med 2013 13 313 317 (in Chinese)

[B6] Zou Y Qi ZQ Feng ML Wang F Li W Li SG Xu ZP Gu JC Clinical analysis of imported *Schistosoma mansoni* infections: a report of two cases and review of the literature Chin Trop Med 2011 11 250 252 (in Chinese)

[B7] Zhou PB Zhou RH Cao CL Clinical observation and nursing of 28 cases with schistosomiasis haematobia Today Nurse 2010 8 37 38 (in Chinese)

[B8] Yi P Yuan LP Wang ZH He YK Jing QS Zhou J Wang HB Li SM Retrospective survey of 184 patients infected with *Schistosoma haematobium* from African countries Chin J Schisto Control 2011 23 441 442 (in Chinese) 22164862

[B9] Morgan JA Dejong RJ Snyder SD Mkoji GM Loker ES *Schistosoma mansoni* and Biomphalaria: past history and future trends Parasitology 2001 123 S211 S228 11769285 10.1017/s0031182001007703

[B10] de Souza CP Cunha Rde C Andrade ZA Development of *Schistosoma mansoni* in *Biomphalaria tenagophila, Biomphalaria straminea and Biomphalaria glabrata* Rev Inst Med Trop Sao Paulo 1995 37 201 206 8525264 10.1590/s0036-46651995000300004

[B11] de Souza CP Jannotti-Passos LK de Freitas JR Degree of host-parasite compatibility between *Schistosoma mansoni* and their intermediate molluscan hosts in Brazil Mem Inst Oswaldo Cruz 1995 90 5 10 10.1590/S0074-02761995000100003 8524084

[B12] Spatz L Cappa SM de Núñez MO Susceptibility of wild populations of *Biomphalaria* spp. from neotropical South America to *Schistosoma mansoni* and interference of *Zygocotyle lunata* J Parasitol 2012 98 1291 1295 10.1645/GE-3002.1 22524265

[B13] Pan SD Chen PJ Rong SM Liu JS Wang JK Chen ZH Zhong JM Investigation on *Biomphalaria straminea*, an intermediate host of *Schistosoma mansoni* in Shenzhen City South Chin J Prev Med 1993 7 70 76 (in Chinese)

[B14] Hotez PJ Fenwick A Schistosomiasis in Africa: An emerging tragedy in our new global health decade PLoS Negl Trop Dis 2009 3 e485 10.1371/journal.pntd.0000485 19787054 PMC2746322

[B15] Xu ZP Chen MG Wang H Song GY Chen RY Yu SH Zhang YQ Yang JS Clinical observations on 67 cases of schistosomiasis mansoni Acta Acad Med Sin 1979 1 127 130 (in Chinese) 262824

[B16] Lu QS Xu LB Analysis of 15 cases of schistosomiasis haematobia J Peking Univ 1980 22 215 216 (in Chinese)

[B17] Feng B Liu YL Han XZ Schistosomiasis haematobia: a report of two cases Shaanxi Med J 1984 13 38 39 (in Chinese)

[B18] Wu ZT E XJ Wang AX Schistosomiasis haematobia: report of 22 cases Acta Acad Med Sin 1988 10 306 307 (in Chinese) 2976322

[B19] Zeng TY Cai YH A case of urinary schistosomiasis Railway Med J 1991 19 382 383 (in Chinese)

[B20] Jin LQ Yi SH Liu Z Chang XH Na WL Wang PX A case of schistosomiasis haematobia J Pathogen Biol 1992 5 III in Chinese

[B21] Hao XH Investigation on two cases infected with *Schistosoma haematobium* Chin J Front Health Quarant 1992 15 340 341 (in Chinese)

[B22] Zeng TY Misdiagnosis of schistosomiasis haematobia as bladder tumor: a case report Clin Misdiagn Misther 1992 5 134 135 (in Chinese)

[B23] Huang LS Analysis of 21 cases with schistosomiasis haematobia Chin J Schisto Control 1992 4 355 (in Chinese)

[B24] Liu J Gan SB Long-term follow-up observation on schistosomiasis mansoni patients Chin J Zoon 2001 17 69 (in Chinese)

[B25] Qian CY Li YZ Xu GY Quantitative observation on eggs in urine of schistosomiasis haematobium treated with praziquantel: one case report Chin J Schisto Control 2005 17 466 467 (in Chinese)

[B26] Lei JC Liu ZX Huang YX An imported case with *Schistosoma haematobium* infection in Angola Chin J Parasitol Parasit Dis 2007 25 I in Chinese 17639711

[B27] Hao Y Zheng H Zhu R Guo JG Wu XH Wang LY Chen Z Zhou XN Schistosomiasis situation in People’s Republic of China in 2008 Chin J Schisto Control 2009 21 451 456 (in Chinese)

[B28] Hao Y Zheng H Zhu R Guo JG Wang LY Chen Z Zhou XN Schistosomiasis situation in People’s Republic of China in 2009 Chin J Schisto Control 2010 22 521 527 (in Chinese)

[B29] Callisto M Moreno P Gonçalves JF Jr Ferreir WR Gomes CLZ Malacological assessment and natural infestation of *Biomphalaria straminea* (Dunker, 1848) by *Schistosoma mansoni* (Sambon, 1907) and *Chaetogaster limnaei* (K. von Baer, 1827) in an urban eutrophic watershed Braz J Biol 2005 65 217 228 16097724 10.1590/s1519-69842005000200005

[B30] Wang YX Zhang WZ The intermediate host of *Schistosoma mansoni*: *Biomphalaria straminea* Chin J Zool 1984 20 18 20 (in Chinese)

[B31] Yang GJ Utzinger J Sun LP Hong QB Vounatsou P Tanner M Zhou XN Effect of temperature on the development of *Schistosoma japonicum* within *Oncomelania hupensis*, and hibernation of *O. hupensis* Parasitol Res 2007 100 695 700 10.1007/s00436-006-0315-8 17031698

[B32] Intergovernmental Panel on Climate Change Climate change 2001: The Scientific Basis 2001 Cambridge: Cambridge University Press

[B33] Qin DH Climate Change: Science, Impact and Countermeasure 2004 Beijing: China Meteorological Press (in Chinese)

[B34] Melman SD Steinauer ML Cunningham C Kubatko LS Mwangi IN Wynn NB Mutuku MW Karanja DM Colley DG Black CL Secor WE Mkoji GM Loker ES Reduced susceptibility to praziquantel among naturally occurring Kenyan isolates of *Schistosoma mansoni* PLoS Negl Trop Dis 2009 3 e504 10.1371/journal.pntd.0000504 19688043 PMC2721635

[B35] Alonso D Muñoz J Gascón J Valls ME Corachan M Failure of standard treatment with praziquantel in two returned travelers with *Schistosoma haematobium* infection Am J Trop Med Hyg 2006 74 342 344 16474094

[B36] Lawn SD Lucas SB Chiodini PL *Schistosoma mansoni* infection: failure of standard treatment with praziquantel in a returned traveler Trans Roy Soc Trop Med Hyg 2003 97 100 101 10.1016/S0035-9203(03)90038-1 12886814

[B37] Katz N Rocha RS de Souza CP Coura Filho P Bruce JI Coles GC Kinoti GK Efficacy of alternating therapy with oxamniquine and praziquantel to treat *Schistosoma mansoni* in children following failure of first treatment Am J Trop Med Hyg 1991 44 509 512 1905880 10.4269/ajtmh.1991.44.509

[B38] Silva IM Thiengo R Conceição MJ Rey L Lenzi HL Pereira Filho E Ribeiro PC Therapeutic failure of praziquantel in the treatment of *Schistosoma haematobium* infection in Brazilians returning from Africa Mem Inst Oswaldo Cruz 2005 100 445 449 16113896 10.1590/s0074-02762005000400018

[B39] Wang W Wang L Liang YS Susceptibility or resistance of praziquantel in human schistosomiasis: a review Parasitol Res 2012 111 1871 1877 10.1007/s00436-012-3151-z 23052781

